# Supplementary material for: Evolution of tissue and developmental specificity of transcription start sites in Bos taurus indicus
Source: Commun Biol. 2021 Jul 1;4:829. doi: 10.1038/s42003-021-02340-6 (PMC8249380; doi:10.1038/s42003-021-02340-6)
Supplement: Supplementary file 1 — Supplementary Information [file 42003_2021_2340_MOESM1_ESM.pdf]

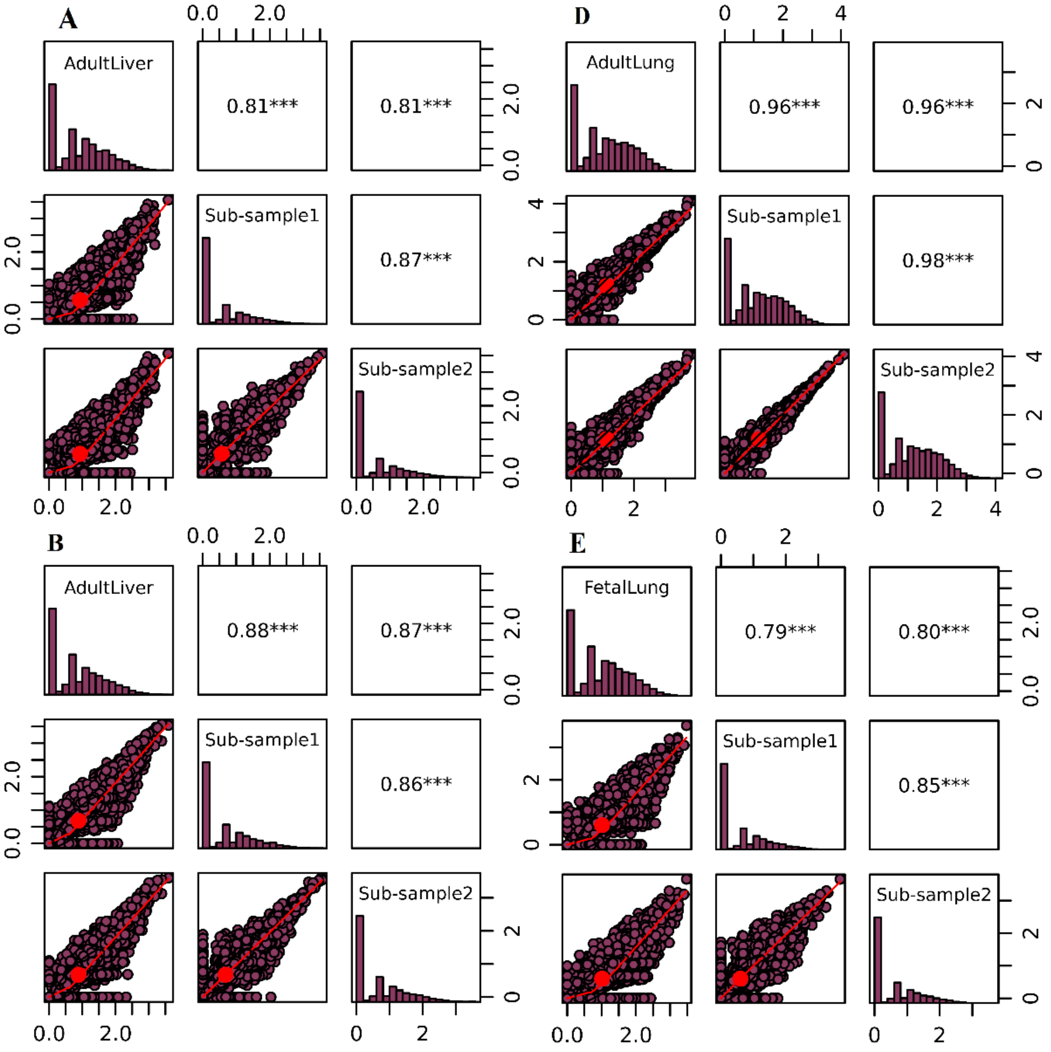

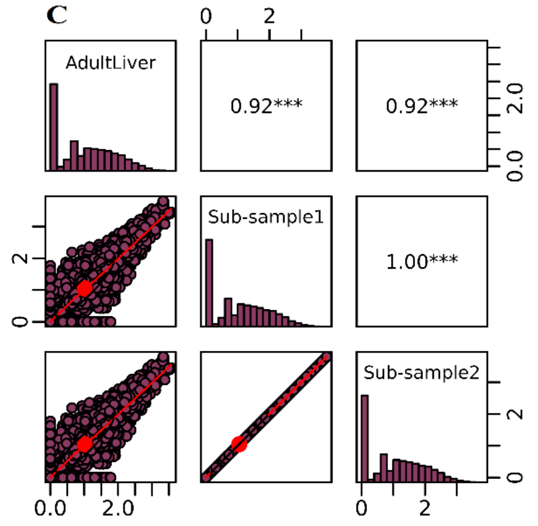

Supplementary Fig. 1. Pearson correlation of TSS diversity measured by Shannon index between total sample and sub-sample1 and 2 in *taurus* adult liver (biological replicate 1, A), *taurus* adult liver (biological replicate 2, B), *indicus* adult liver (C), *indicus* adult lung (D), and *indicus* fetal lung (E) tissues. The distribution of Shannon index is shown on the diagonal and on the bottom of the diagonal the bivariate scatter plots with a fitted line are displayed. Also on the top of the diagonal, the value of the correlation plus the significance level (P-value<0.001) as stars is shown

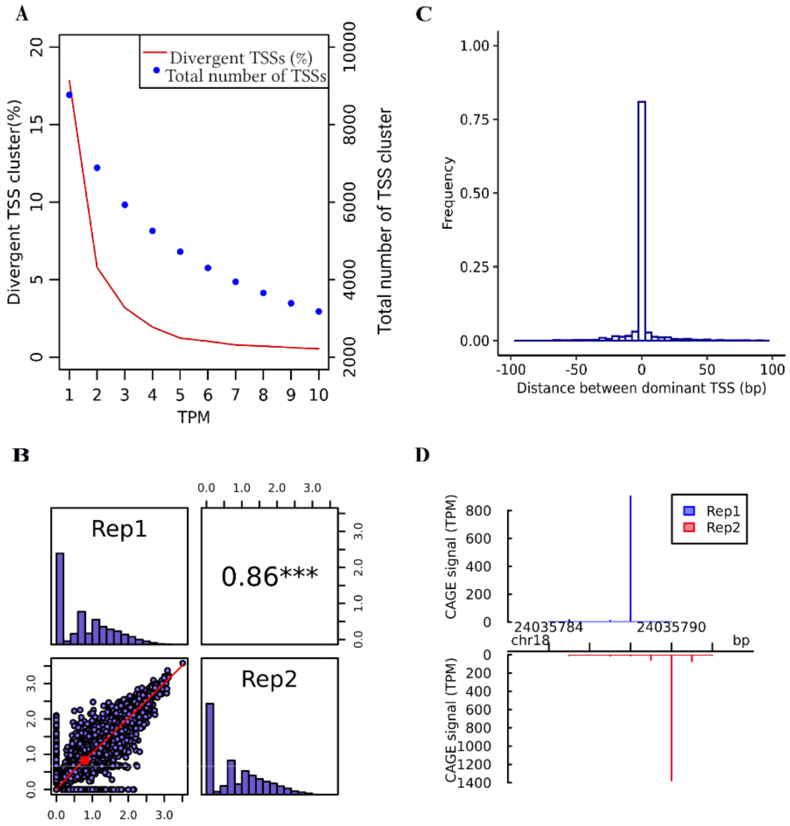

Supplementary Fig. 2. Overview of similarity between *Bos taurus* biological replicates in adult liver. A) Total number of TSS clusters and their percentage of divergent TSS at different expression levels (TPM), B) Pearson correlation of TSS diversity measured by Shannon index (the distribution of Shannon index is shown on the diagonal and on the bottom of the diagonal the bivariate scatter plot with a fitted line are displayed. Also on the top of the diagonal, the value of the correlation plus the significance level (P-value<0.001) as stars is shown), C) Histogram of distances between dominant TSS of *Bos taurus* biological replicates in consensus TSS cluster with at least 10 TPM level expression, and D) An example of differential TSS usage observed in gene *MT1E* across biological replicates (P-value and FDR<0.05; shifting score=0.97). The annotated TSS based on the Ref-Seq gene using Apr.2018 (ARS-UCD1.2/bosTau9) is located at position 24,035,790 bp (NM_001114857) metallothionein 1E.

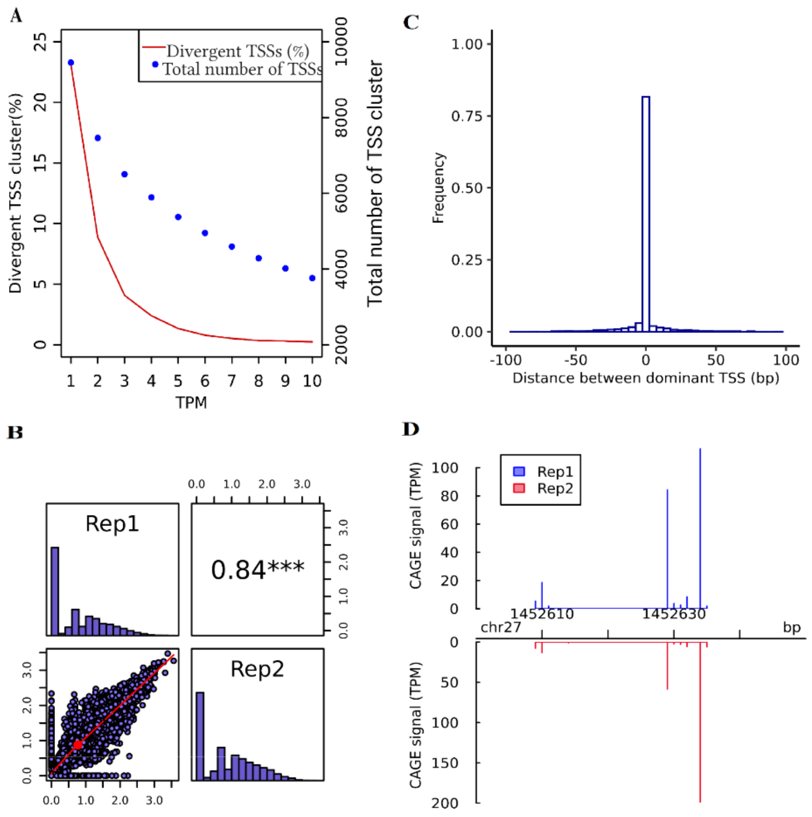

Supplementary Fig. 3. Overview of similarity between *Bos taurus* biological replicates in adult muscle. A) Total number of TSS clusters and their percentage of divergent TSS at different expression levels (TPM), B) Pearson correlation of TSS diversity measured by Shannon index (the distribution of Shannon index is shown on the diagonal and on the bottom of the diagonal the bivariate scatter plot with a fitted line are displayed. Also on the top of the diagonal, the value of the correlation plus the significance level (P-value<0.001) as stars is shown), C) Histogram of distances between dominant TSS of *Bos taurus* biological replicates in consensus TSS cluster with at least 10 TPM level expression, and D) An example of differential TSS usage observed in gene *MYOM2* across biological replicates (P-value and FDR<0.05; shifting score=0.14). The annotated TSS based on the Ref-Seq gene using Apr.2018 (ARS-UCD1.2/bosTau9) is located at position 1,452,630 bp – (NM_001038140) myomesin-2

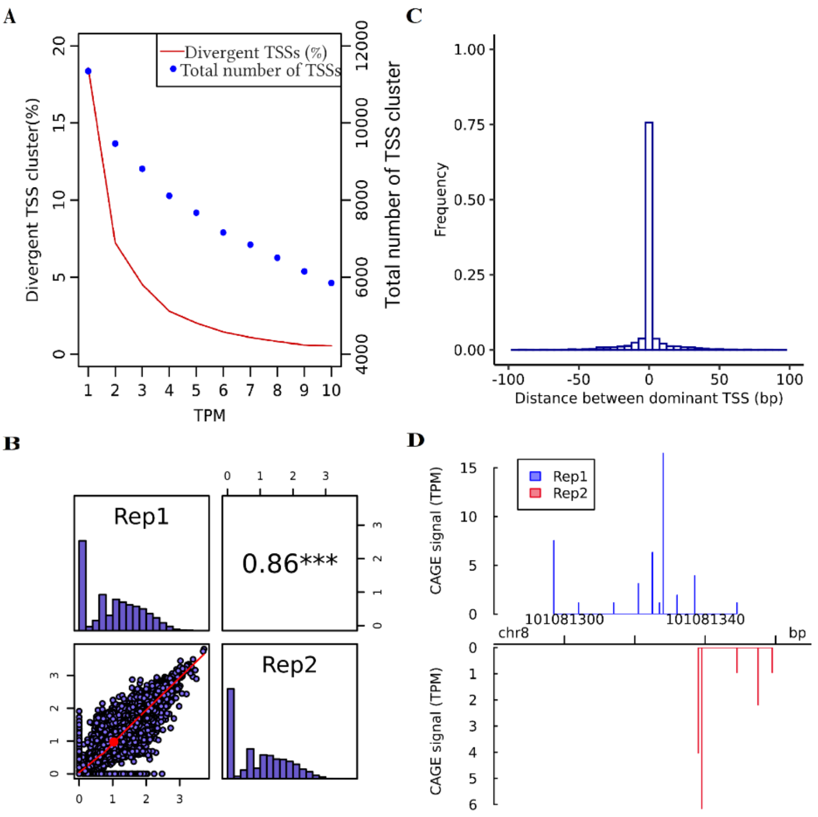

Supplementary Fig. 4. Overview of similarity between *Bos taurus* biological replicates in adult spleen. A) Total number of TSS clusters and their percentage of divergent TSS at different expression levels (TPM), B) Pearson correlation of TSS diversity measured by Shannon index (the distribution of Shannon index is shown on the diagonal and on the bottom of the diagonal the bivariate scatter plot with a fitted line are displayed. Also on the top of the diagonal, the value of the correlation plus the significance level (P-value<0.001) as stars is shown), *C)* Histogram of distances between dominant TSS of *Bos taurus* biological replicates in consensus TSS cluster with at least 10 TPM level expression, and D) An example of differential TSS usage observed in gene *GNG10* across biological replicates (P-value and FDR<0.05; shifting score=0.91). The annotated TSS based on the Ref-Seq gene using Apr.2018 (ARS-UCD1.2/bosTau9) is located at position 101,081,328 bp - (NM_001114512) guanine nucleotide-binding protein G(I)/G(S)/G(O) subunit gamma-10

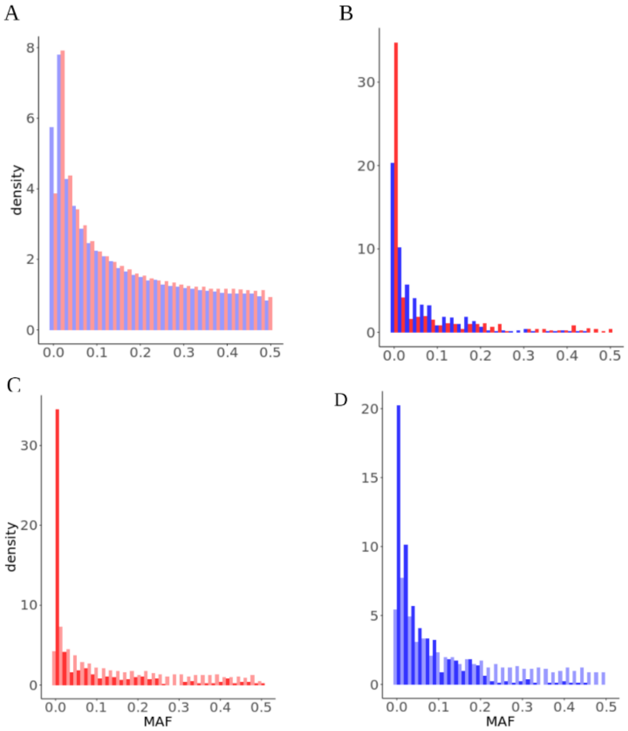

Supplementary Fig. 5. Distribution of minor allele frequency (MAF) across *Bos taurus* (Red colour) and *Bos indicus* (Blue colour) sub-species. **A**. MAF is calculated for all genome wide SNPs available in 1000 bull genomes project (run 8) with MAF>0.005. **B**. MAF is calculated for the significant SNPs with a significant shift in allele frequency within differentially used TSS regions across sub-species. **C**. Probability distribution of MAF in a sample of 472 SNPs drawn from *Bos taurus* genome-wide SNPs (light red) in comparison with MAF distribution for the significant SNPs within differentially used TSS regions in *Bos taurus* sub-species (dark red). **D**. Probability distribution of MAF in a sample of 472 SNPs drawn from *Bos indicus* genome-wide SNPs (light blue) in comparison with MAF distribution for the significant SNPs within differentially used TSS regions in *Bos indicus* sub-species (dark blue).

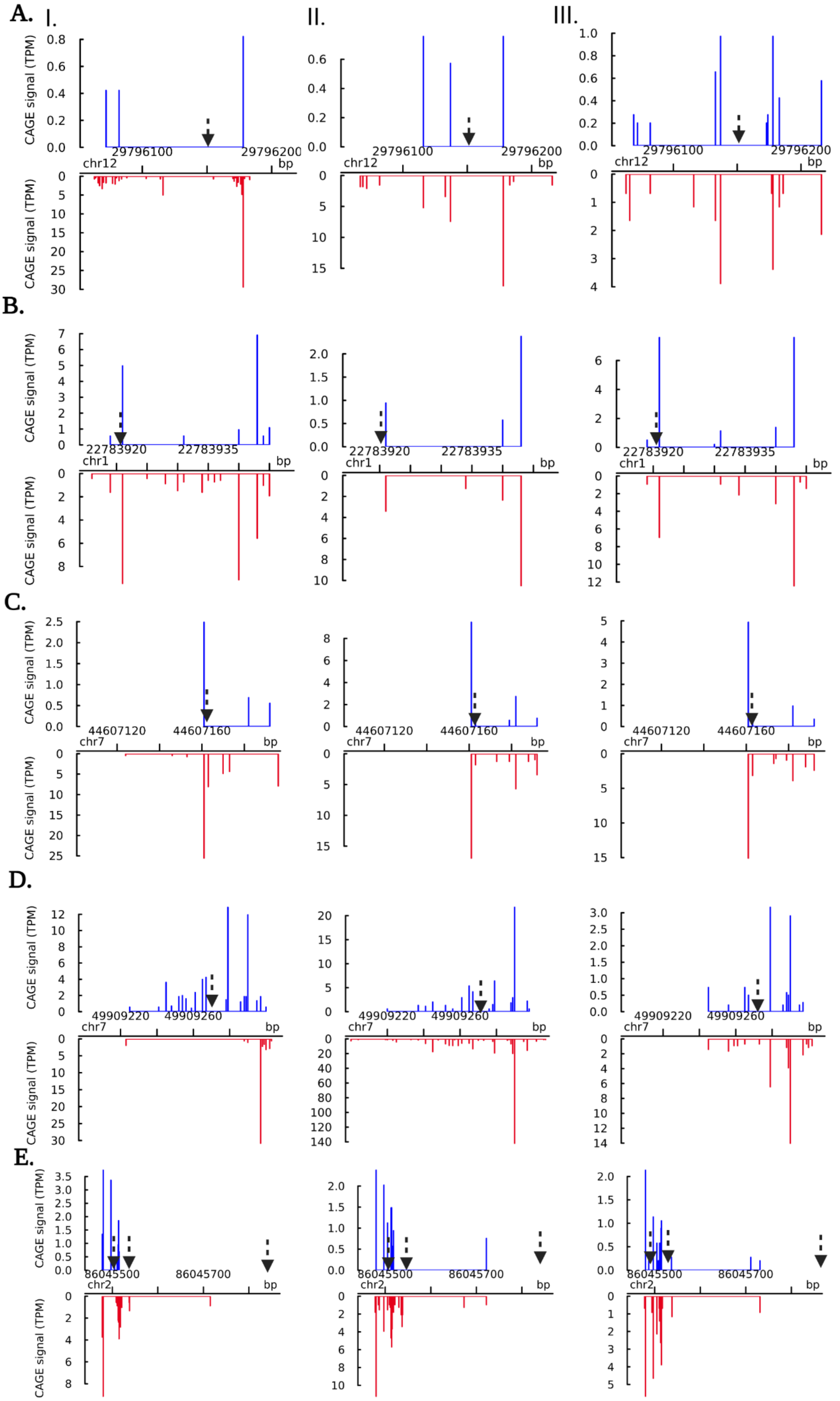

Supplementary Fig. 6. Distribution of CAGE tags among the consensus TSS cluster in *taurus* and *indicus* sub-species (Blue and red colours, respectively) in heat shock protein related genes *(*A.** ***HSPH1*****, B.** ***HSPA13*****, C.** ***HSPA4*****, D.*****HSPA9*****, E.** ***HSPD1*****) in liver (i), muscle (ii), and spleen (iii) tissues.** The annotated TSS (dashed arrow) for *HSPH1*, *HSPA13*, *HSPA4*, *HSPA9* based on the Ref-Seq gene (using ARS-UCD1.2/bosTau9 Apr.2018) are located at position (29,796,159), (22,783,919), (44,607,161), (49,909,267) and for gene *HSPD1* at positions (86,045,521; 86,045,870; 86,045,509), respectively.

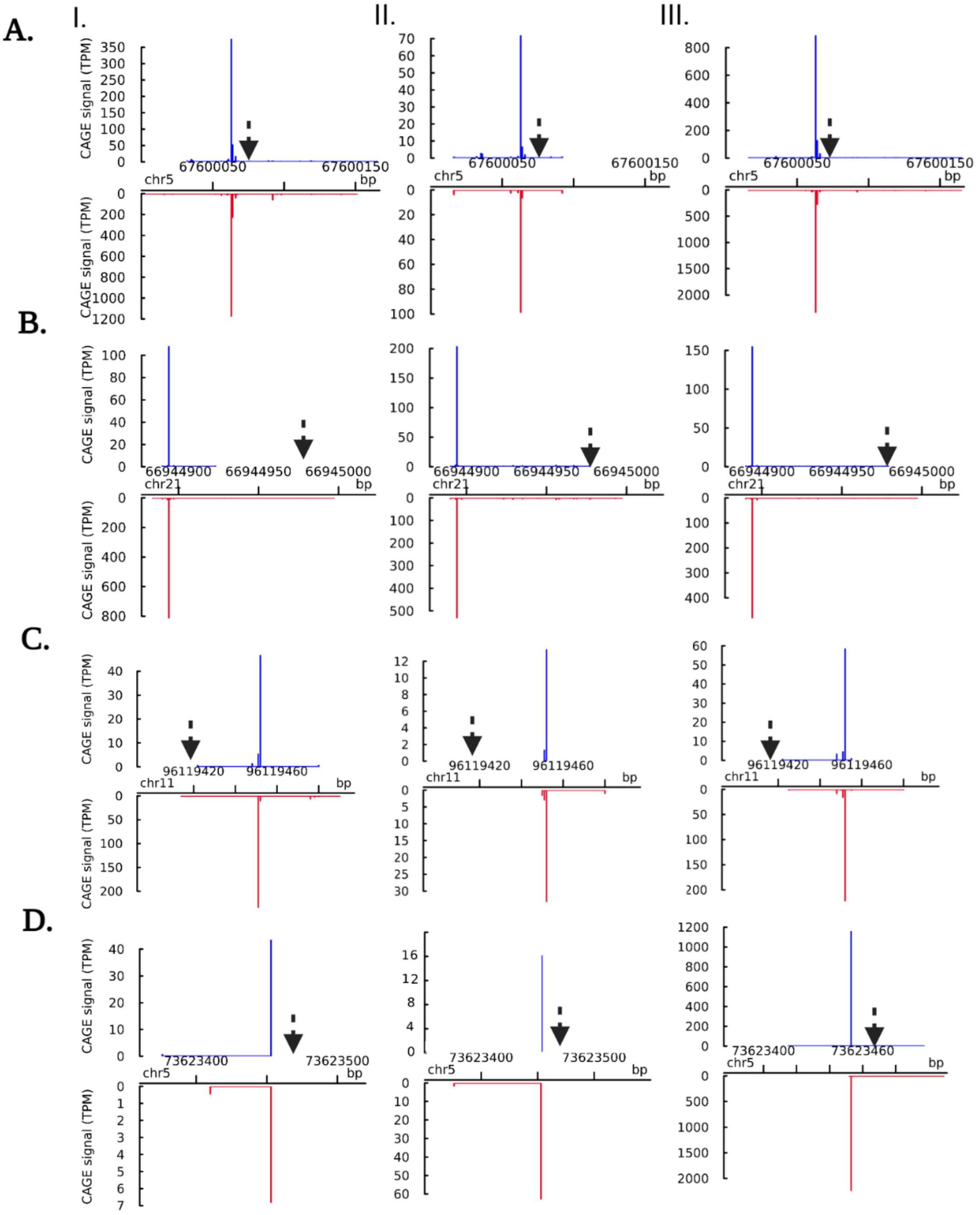

Supplementary Fig. 7. Distribution of CAGE tags among the consensus TSS cluster in *taurus* and *indicus* sub-species (Blue and red colours, respectively) in heat shock protein related genes (A. *HSP90B1*, B. *HSP90AA1*, C. *HSPA5*, D. *HMOX1*) in liver (i), muscle (ii), and spleen (iii) tissues. The annotated TSS (dashed arrow) for *HSP90B1*, *HSP90AA1*, *HSPA5*, *HMOX1* based on the Ref-Seq gene (using ARS-UCD1.2/bosTau9 Apr.2018) are located at position (67,600,075), (66,944,979), (96,119,415), (73,623,470), respectively.

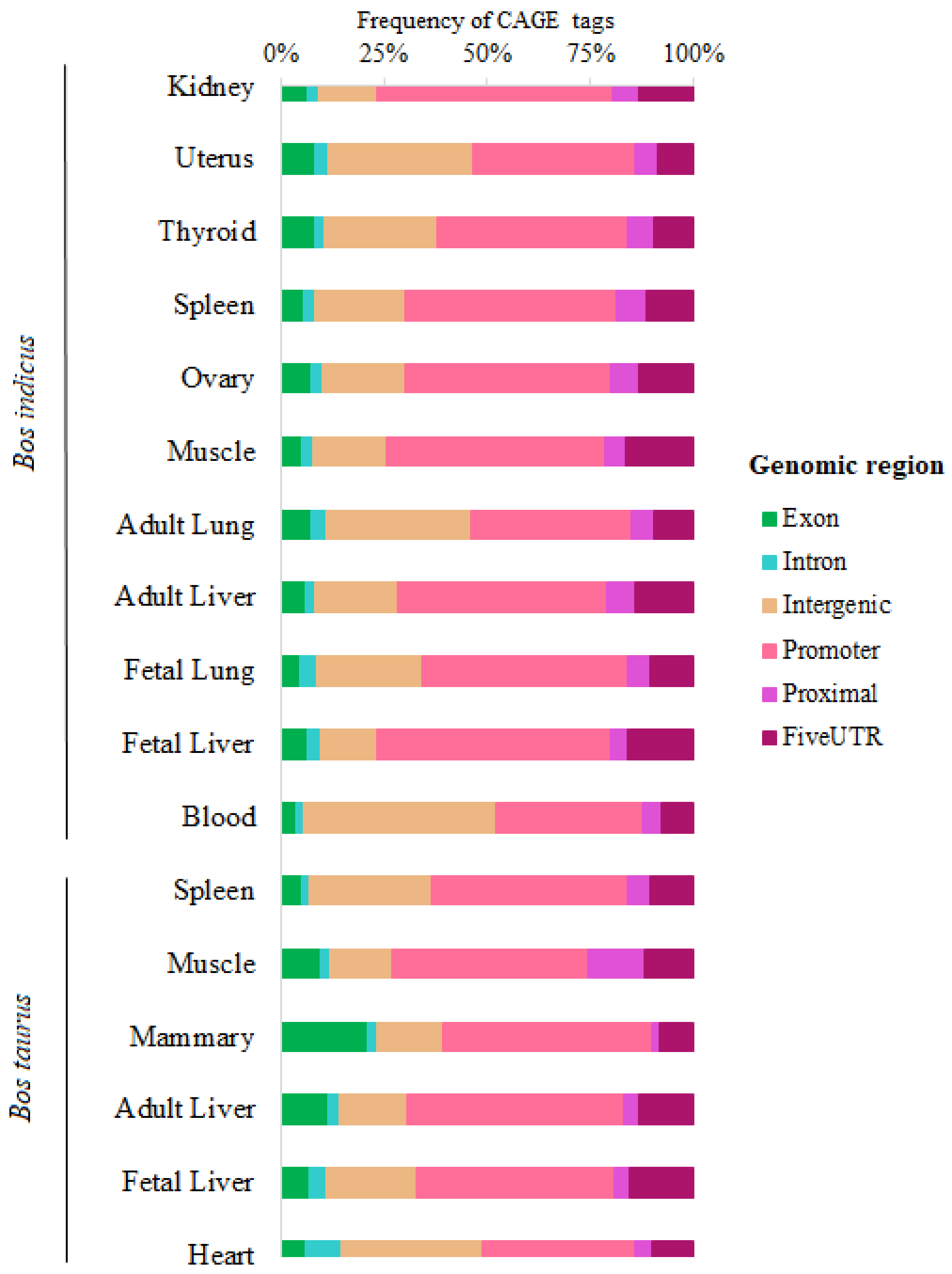

Supplementary Fig. 8. Distribution of the CAGE tags in the diverse type of genomic regions for all of the tissues investigated in *Bos taurus* and *Bos indicus.* Annotation with ENSEMBL Bos taurus ARS-UCD1.2 (gene, transcripts and exon coordinates).

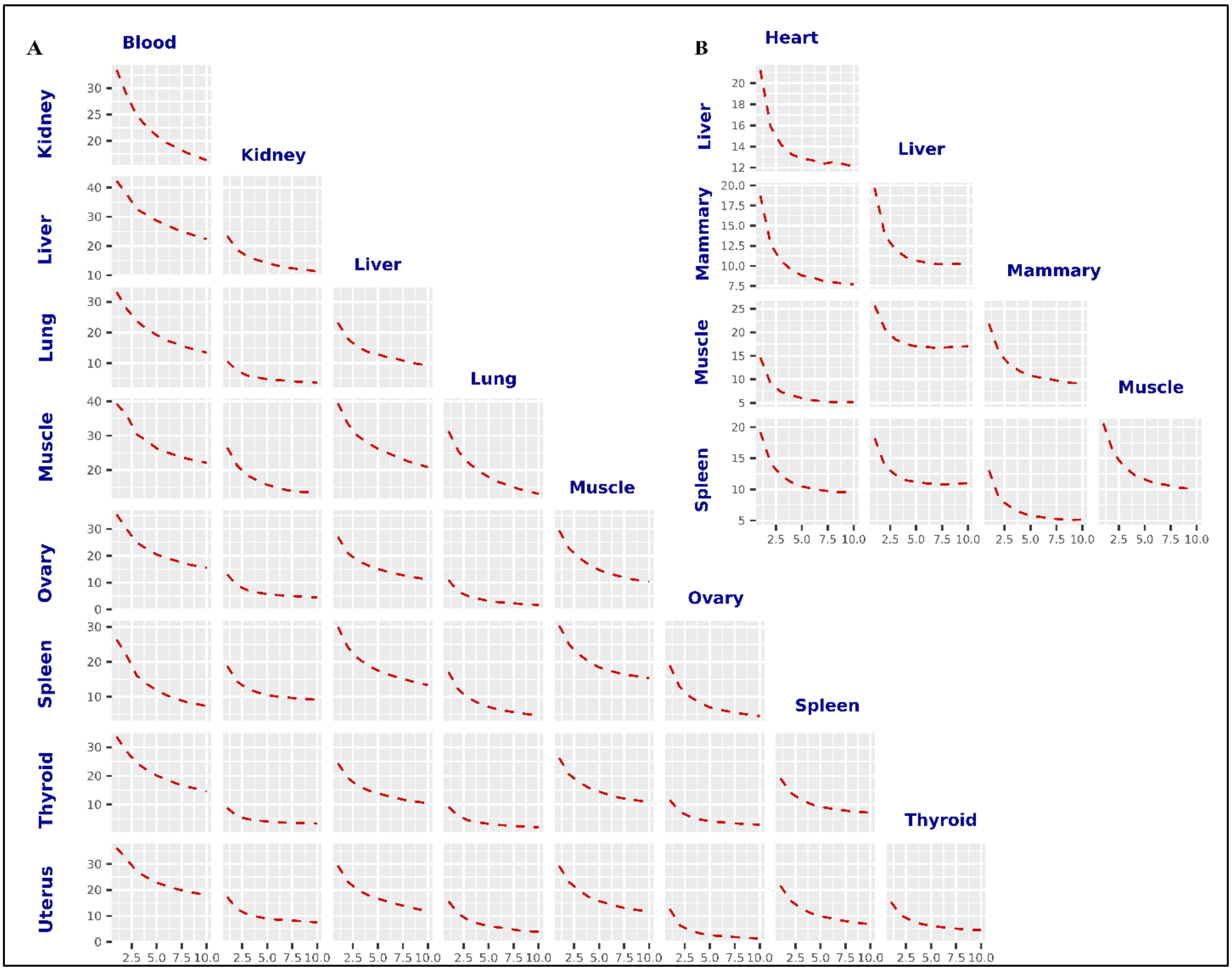

Supplementary Fig. 9. Frequency (%) of divergent TSS clusters (x-axis) at different expression level (TPM; y-axis) across adult tissues in *Bos indicus* (A) and *Bos taurus* sub-species (B).

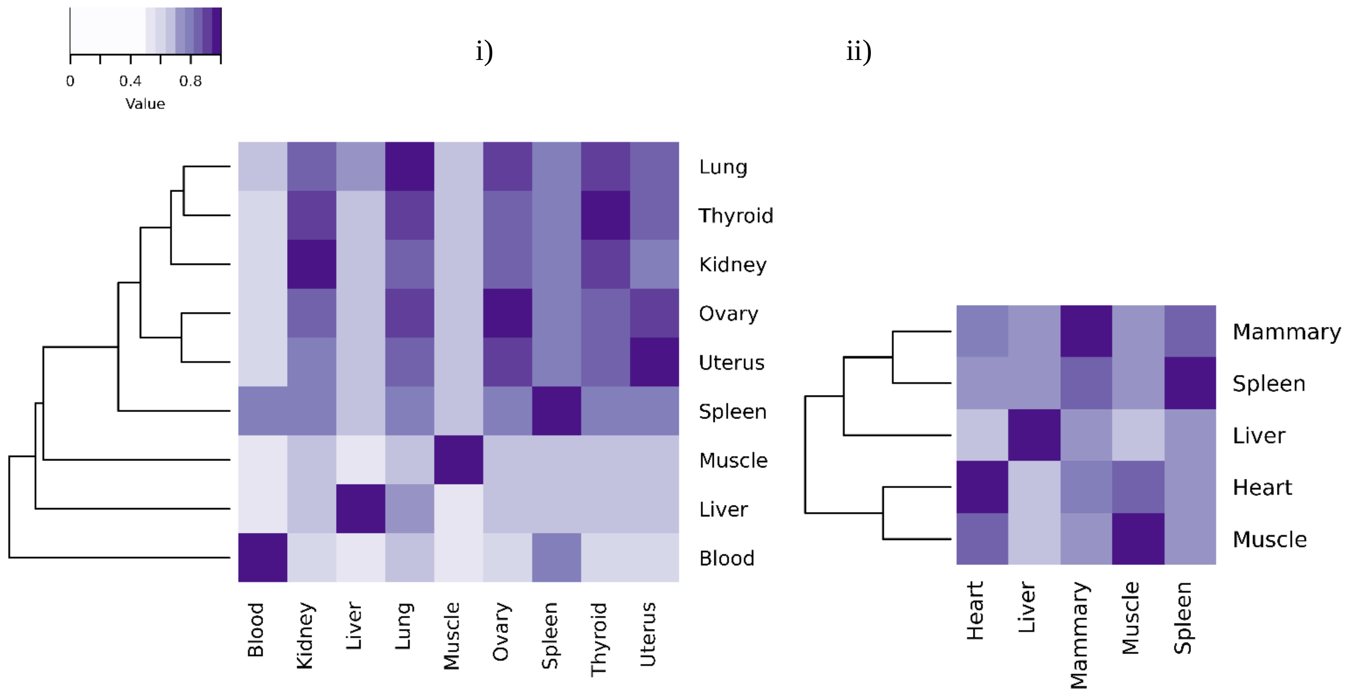

Supplementary Fig. 10. Pearson correlation between Shannon index of TSS diversity between adult tissues in *Bos indicus* (i) and *Bos taurus* (ii).

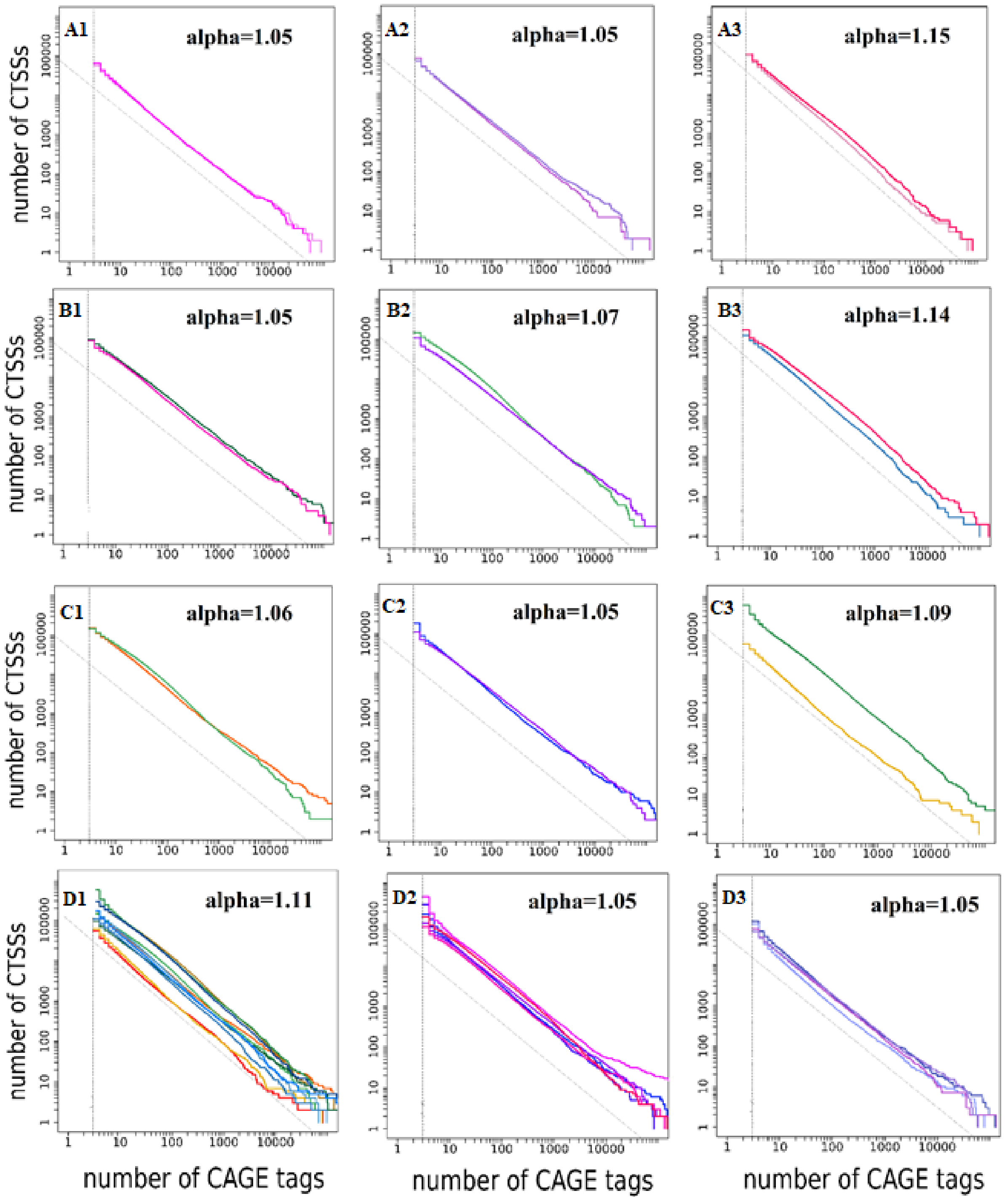

Supplementary Fig. 11. Reverse cumulative distributions for the number of different CTSS positions that have at least a given number of tags mapping to them. Both axes are shown on a logarithmic scale. A1-3 correspond to the biological replicates in muscle, liver, and spleen, respectively. B1-3 correspond to *Bos taurus* and *Bos indicus* muscle, liver and spleen, respectively. C1-3 represent the distribution for *Bos indicus* fetal and adult liver, *Bos taurus* fetal and adult liver, *Bos indicus* fetal and adult lung, respectively. D1-2 correspond to the all *Bos indicus* and *Bos taurus* tissues, respectively, and D3 correspond to *Bos taurus* fetal and adult liver replicates. The value of alpha is slope of the suggested reference distribution for normalization.

Supplementary Table 1. Summary of the coverage of bam files, number of CAGE tags after quality control and number of CTSS for each sample along with the biological replicate.

Tissue	Biological replicate	Total number of reads in bam file	Total number of CAGE tags after quality control	Number of CAGE tags after filtering (CTSS<3tags)	Number of CTSS		Number of TSSs
					In sample	Across all replicates	
Bos indicus							
Adult stage							
Blood	1	4,986,447	2,461,582	992,298	54,150	-	21,234
Kidney	1	20,244,459	12,568,610	9,551,097	286,988	-	21,443
Muscle	1	7,414,926	4,565,131	3,477,025	92,772	-	18,404
Ovary	1	12,315,069	5,631,882	3,668,458	164,737	-	22,304
Uterus	1	12,211,715	6,884,223	3,918,297	172,668	-	27,063
Spleen	1	6,164,132	3,329,665	2,441,270	108,189	-	17,965
Thyroid	1	18,954,871	10,541,125	8,857,443	292,047	-	22,452
Liver	1	19,113,216	5,183,691	4,615,805	144,667	-	20,486
Lung	1	37,473,013	22,195,598	11,603,659	564,140	-	26,878
Fetal stage							
Liver	1	15,141,599	7,448,786	5,478,224	150,202	-	26,389
Lung	1	2,944,730	1,507,191	1,190,439	60,025	-	18,323
Bos taurus							
Adult stage							
Heart	1	18,413,262	11,062,868	1,808,947	169,941	302,705	23,591
	2	19,046,955	10,984,678	1,771,181	194,320		
Liver	1	10,006,006	5,850,606	1,760,207	79,952	107,750	13,144
	2	9,556,940	5,678,632	2,067,870	68,065		
Mammary	1	17,144,293	10,537,485	2,526,355	93,779	470,959	22,758
	2	34,387,398	20,215,087	5,151,462	278,663		
	3	12,600,429	7,777,045	2,708,421	67,919		
	4	25,692,124	16,379,024	5,972,616	193,331		
Muscle	1	6,954,552	3,682,551	1,451,759	56,430	86,649	13,870
	2	6,657,969	3,883,885	1,400,293	66,136		
Spleen	1	14,223,084	7,209,184	1,771,696	98,542	149,867	12,997
	2	17,751,506	8,789,692	2,382,557	108,296		
Fetal stage							
Liver	1	18,212,583	10,048,108	2,477,600	119,656	178,317	17,180
	2	16,615,305	9,327,767	1,509,995	105,333		

Supplementary Table 2. Comparison between the number of consensus TSS clusters in *Bos taurus* adult liver and *Bos indicus* adult liver and lung, and fetal lung samples (total sample) and the half sample (sub-sample 1)

Tissue		Number of CAGE tags after quality control	Number of CAGE tags after filtering (CTSS<3tags)	Number of consensus TSS cluster	Number of consensus TSS cluster in promoter region	Number of consensus TSS cluster in promoter of known gene
Bos taurus Adult Liver						
Biological replicate1	Total sample	5,850,606	1,760,207	*31,213*	9,121	7,082
	Sub-sample1	2,925,099	811,820	*12,135*	6,642	5,366
	Sub-sample2	2,925,506	811,535	12,054	6,553	5,314
Biological replicate2	Total sample	5,678,632	2,067,870	12,007	7,827	6,186
	Sub-sample1	2,839,380	979,767	9,590	6,512	5,264
	Sub-sample2	2,839,251	979,723	9,588	6,512	5,259
Bos indicus Adult Liver						
Biological replicate1	Total sample	5,183,691	4,615,805	17,188	10,930	8,428
	Sub-sample1	2,591,859	2,209,032	16,862	10,783	8,398
	Sub-sample2	2,591,831	2,209,206	16,991	10,851	8,377
Adult Lung						
Biological replicate1	Total sample	22,195,598	11,603,659	19,312	10,713	8,558
	Sub-sample1	11,097,802	5,324,686	19,778	10,670	8,539
	Sub-sample2	11,097,795	5,330,816	19,754	10,652	8,520
Fetal Lung						
Biological replicate1	Total sample	1,507,191	1,190,439	13,200	9,346	7,204
	Sub-sample1	753,642	546,026	8,933	6,862	5,535
	Sub-sample2	753,548	545,338	8,946	6,904	5,564

Supplementary Table 3. Comparison of consensus TSS clusters between *Bos taurus* biological replicates1 and 2 in three adult tissues (spleen, liver and muscle)

Tissue	Number of consensus clusters across both replicates		Number of consensus TSS clusters with at least 10 TPM expression level		Number of divergent TSSs [1]	Number of significant TSSs with differential usage [1] and shifting score >0.1	
	Total number	In promoter regions	Total number	In promoter regions		Total number	Significant (FDR P-value<0.05)
Spleen	35,874	11,343	7,356	5,851	32	1660	27
Liver	16,673	8,768	4,035	3,178	17	581	9
Muscle	29,001	9,453	4,767	3,760	9	1140	11

[1] with at least 10 TPM expression level in promoter region

Supplementary Table 4. HSP family related genes

Gene symbol	Symbol	Full gene name
HSP32	HMOX1	heme oxygenase 1 3.7
HSP60	HSPD1	heat shock protein family D (Hsp60) member 1 2.6
HSP70	HSPA4	heat shock protein family A (Hsp70) member 4 3.5
	HSPA5	heat shock protein family A (Hsp70) member 5 3.1
	HSPA9	heat shock protein family A (Hsp70) member 9 3.7
	HSPA13	heat shock protein family A (Hsp70) member 13 4.9
	HSPA14	heat shock protein family A (Hsp70) member 14 3.0
HSP90	HSP90AA1	heat shock protein 90 kDa alpha family class A member 1 2.6
	HSP90B1	heat shock protein 90 kDa beta family member 1 3.2
HSP105	HSPH1	heat shock 105 kDa/110 kDa protein 1 3.7
