# Supplementary material for: Evolution of tissue and developmental specificity of transcription start sites in Bos taurus indicus
Source: Commun Biol. 2021 Jul 1;4:829. doi: 10.1038/s42003-021-02340-6 (PMC8249380; doi:10.1038/s42003-021-02340-6)
Supplement: Supplementary file 15 — Description of Supplementary Files [file 42003_2021_2340_MOESM15_ESM.pdf]

Description of Additional Supplementary Files

File name: Supplementary Data 1
Description: Source TSSs data in adult liver tissue across taurus and indicus subspecies.

File name: Supplementary Data 2
Description: Source TSSs data in spleen tissue across *taurus* and *indicus* subspecies.

File name: Supplementary Data 3
Description: Source TSSs data muscle tissue across *taurus* and *indicus* subspecies.

File name: Supplementary Data 4
Description: List of genes with significant differential TSS usage with shifting score >0.1 across Bos taurus biological replicates in A) muscle, B) spleen, and C) liver tissues (P-value<0.05).

File name: Supplementary Data 5
Description: List of genes with significant differential TSS usage with shifting score >0.1 across Bos indicus and Bos taurus sub-species in A) muscle, B) spleen, and C) liver tissues (P-value<0.05).

File name: Supplementary Data 6
Description: Significant gene ontology (GO) terms for genes with significant differential TSS usage with shifting score >0.1 across Bos taurus and Bos indicus sub-species and Bos taurus biological replicates in muscle, liver, and spleen (FDR P-value <0.05). Circle pattern shows the fold enrichment; (>50%), (25-50%), (12.5-25%) and (<12.5%) values are red, yellow, green, and grey, respectively.

File name: Supplementary Data 7
Description: Genotype, minor allele frequencies (MAF) of SNPs among significant differential consensus TSS clusters with a significant shift in allele frequency across sub-species, identified using a simple FST-based method (2N= the sum of genotyped gametes in the two populations, and **FDR P-value<0.005 and *** FDR P-value<0.001).

File name: Supplementary Data 8

Description: Source TSSs data in *taurus* liver tissue across developmental stage.

File name: Supplementary Data 9
Description: Source TSSs data in *indicus* liver tissue across developmental stage.

File name: Supplementary Data 10
Description: List of genes with significant differential TSS usage with shifting score >0.1 across fetal and adult stages in Bos indicus liver, Bos taurus liver, and Bos indicus lung (P-value<0.05).

File name: Supplementary Data 11
Description: Significant gene ontology (GO) terms for genes with significant differential TSS usage with shifting score >0.1 across fetal and adult stages in Bos indicus liver, Bos taurus liver, and Bos indicus lung (FDR P-value <0.05). Circle pattern shows the fold enrichment; (>50%), (25-50%), (12.5-25%) and (<12.5%) values are red, yellow, green, and grey, respectively.

File name: Supplementary Data 12
Description: List of genes with significant cross switching TSS event across fetal and adult stages in Bos taurus liver (P-value<0.05).

File name: Supplementary Data 13
Description: Source TSSs data in *indicus* lung tissue across developmental stage
