# Supplementary material for: Evolution of tissue and developmental specificity of transcription start sites in Bos taurus indicus
Source: Commun Biol. 2021 Jul 1;4:829. doi: 10.1038/s42003-021-02340-6 (PMC8249380; doi:10.1038/s42003-021-02340-6)
Supplement: Supplementary file 17 — Reporting Summary [file 42003_2021_2340_MOESM17_ESM.pdf]

nature research

Corresponding author(s): Mehrnush Forutan

Last updated by author(s): May 24, 2021

Reporting Summary

Nature Research wishes to improve the reproducibility of the work that we publish. This form provides structure for consistency and transparency in reporting. For further information on Nature Research policies, see our Editorial Policies and the Editorial Policy Checklist.

Statistics

For all statistical analyses, confirm that the following items are present in the figure legend, table legend, main text, or Methods section.

n/a	Confirmed	
☐	☒	The exact sample size (n) for each experimental group/condition, given as a discrete number and unit of measurement
☐	☒	A statement on whether measurements were taken from distinct samples or whether the same sample was measured repeatedly
☐	☒	The statistical test(s) used AND whether they are one- or two-sided<br>*Only common tests should be described solely by name; describe more complex techniques in the Methods section.*
☐	☒	A description of all covariates tested
☐	☒	A description of any assumptions or corrections, such as tests of normality and adjustment for multiple comparisons
☐	☒	A full description of the statistical parameters including central tendency (e.g. means) or other basic estimates (e.g. regression coefficient) AND variation (e.g. standard deviation) or associated estimates of uncertainty (e.g. confidence intervals)
☐	☒	For null hypothesis testing, the test statistic (e.g. F, t, r) with confidence intervals, effect sizes, degrees of freedom and P value noted<br>*Give P values as exact values whenever suitable.*
☒	☐	For Bayesian analysis, information on the choice of priors and Markov chain Monte Carlo settings
☐	☒	For hierarchical and complex designs, identification of the appropriate level for tests and full reporting of outcomes
☐	☒	Estimates of effect sizes (e.g. Cohen's d, Pearson's r), indicating how they were calculated

Our web collection on statistics for biologists contains articles on many of the points above.

Software and code

Policy information about availability of computer code

Data collection	No software was used
Data analysis	QubitTM 3.0 and 4.0 Fluorometer and the Nanodrop ND-1000 spectrophotometer (v.3.5.2, Thermo Fisher Scientific)<br>samtools (version 0.1.19)<br>FastQC<br>Trimmomatic (version 0.35)<br>Burrows-Wheeler Aligner (BWA, version 0.7.13)<br>Bowtie 2(version 2/2.3.4.3)<br>dwgsim<br>CAGEr<br>DAVID8<br>R 4.0.2<br>VCFtools(version 0.1.13)

For manuscripts utilizing custom algorithms or software that are central to the research but not yet described in published literature, software must be made available to editors and reviewers. We strongly encourage code deposition in a community repository (e.g. GitHub). See the Nature Research guidelines for submitting code & software for further information.

Data

Policy information about availability of data

All manuscripts must include a data availability statement. This statement should provide the following information, where applicable:

- Accession codes, unique identifiers, or web links for publicly available datasets
- A list of figures that have associated raw data
- A description of any restrictions on data availability

Bos taurus and Bos indicus raw sequence data are publicly available via European Nucleotide Archive (ENA) under study ID PRJEB43513 and PRJEB44817, respectively. Sample metadata for Bos taurus is available in the BioSamples database under accessions SAMEA8326848, SAMEA8326850, SAMEA4447839, SAMEA4447825, SAMEA4447799 and SAMEA4447832. Sample metadata for Bos indicus is available in the BioSamples database under accessions SAMEA8976600, SAMEA8976601, SAMEA8976602, SAMEA8976603, SAMEA8976604, SAMEA8976605, SAMEA8976606, SAMEA8976607, SAMEA8976608, SAMEA8976609, SAMEA8976610. All other relevant data are available in this article and its Supplementary Information files. See Supplementary Table 1–4, Supplementary Data 1-7, and Supplementary Fig. 1-11 for extended biological findings, and Supplementary Data 8-13 for Fig. 1-6 and Table 1-2 source data.

Field-specific reporting

Please select the one below that is the best fit for your research. If you are not sure, read the appropriate sections before making your selection.

- [x] Life sciences
- [ ] Behavioural & social sciences
- [ ] Ecological, evolutionary & environmental sciences

For a reference copy of the document with all sections, see nature.com/documents/nr-reporting-summary-flat.pdf

Life sciences study design

All studies must disclose on these points even when the disclosure is negative.

Sample size	Sample size was determined by the availability of the tissues and one Brahman adult and fetus, and four Holstein lactating cows, including two pregnant (16 weeks gestation).
Data exclusions	We did not any data exclusion.
Replication	Due to the low number of samples available a power estimation was undertaken by splitting the data into lower depth and determining if the same calls were made. The subspecies differences are expected to be large in comparison to within species variation. Biological replication included total 5 adult cattle were used and 3 cattle fetus.
Randomization	Holstein animals were randomly selected from the herd. The Brahman animal was selected based on pedigree and availability (she was being commercially slaughtered).
Blinding	There was no blinding at any stage of the study/analysis.

Reporting for specific materials, systems and methods

We require information from authors about some types of materials, experimental systems and methods used in many studies. Here, indicate whether each material, system or method listed is relevant to your study. If you are not sure if a list item applies to your research, read the appropriate section before selecting a response.

Materials & experimental systems

n/a	Involved in the study	
☒	☐	Antibodies
☒	☐	Eukaryotic cell lines
☒	☐	Palaeontology and archaeology
☐	☒	Animals and other organisms
☒	☐	Human research participants
☒	☐	Clinical data
☒	☐	Dual use research of concern

Methods

n/a	Involved in the study	
☒	☐	ChIP-seq
☒	☐	Flow cytometry
☒	☐	MRI-based neuroimaging

Animals and other organisms

Policy information about studies involving animals; ARRIVE guidelines recommended for reporting animal research

Laboratory animals	The study did not involve the laboratory animals.
Wild animals	The study did not involve the wild animals.

Field-collected samples	For the Brahman animals: Samples were collected from post-slaughter area of a commercial abattoir, no live animals were involved in the study<br>For the Holstein animals: Four lactating cows, two of which were pregnant (16 weeks gestation) were selected from the Agriculture Victoria Research dairy herd at Ellinbank and euthanized by veterinarian
Ethics oversight	The DEDJTR Animal Ethics Committee (2014-23) and the Queensland Department of Agriculture and Fisheries Animal Ethics Committee

Note that full information on the approval of the study protocol must also be provided in the manuscript.
